# Supplementary material for: Lipids and Fatty Acid Composition Reveal Differences between Durum Wheat Landraces and Modern Cultivars
Source: Plants (Basel). 2024 Jul 1;13(13):1817. doi: 10.3390/plants13131817 (PMC11244281; doi:10.3390/plants13131817)

LIPIDOGRAM METHOD

Example of overlapping of all standard chromatograms with matrix (sample KRONOS).

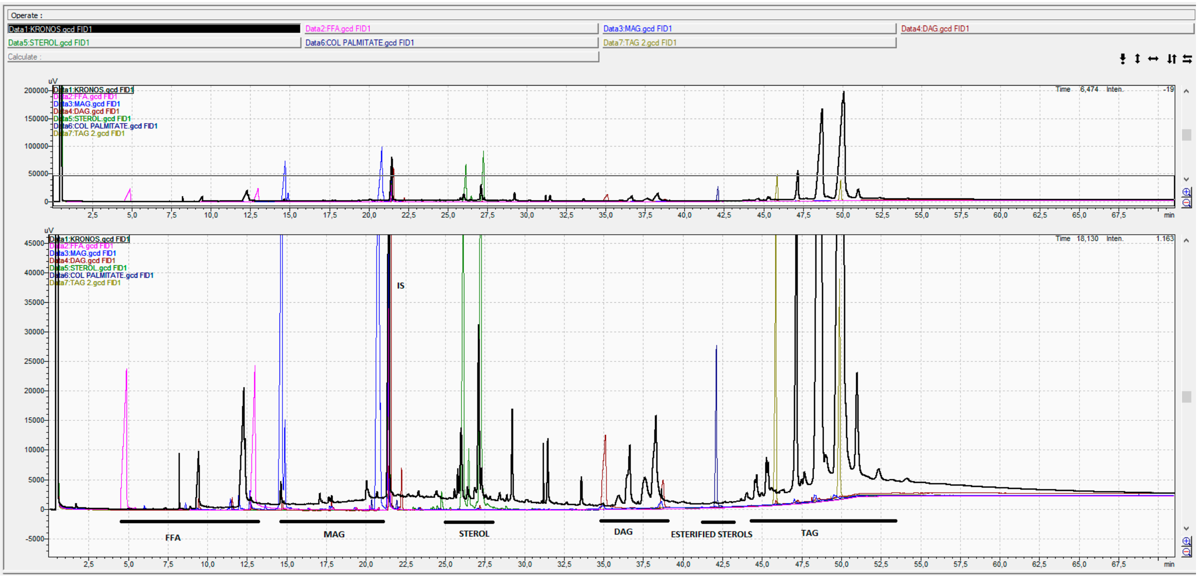

Example of overlapping of all standard chromatograms with matrix (sample AUREO).

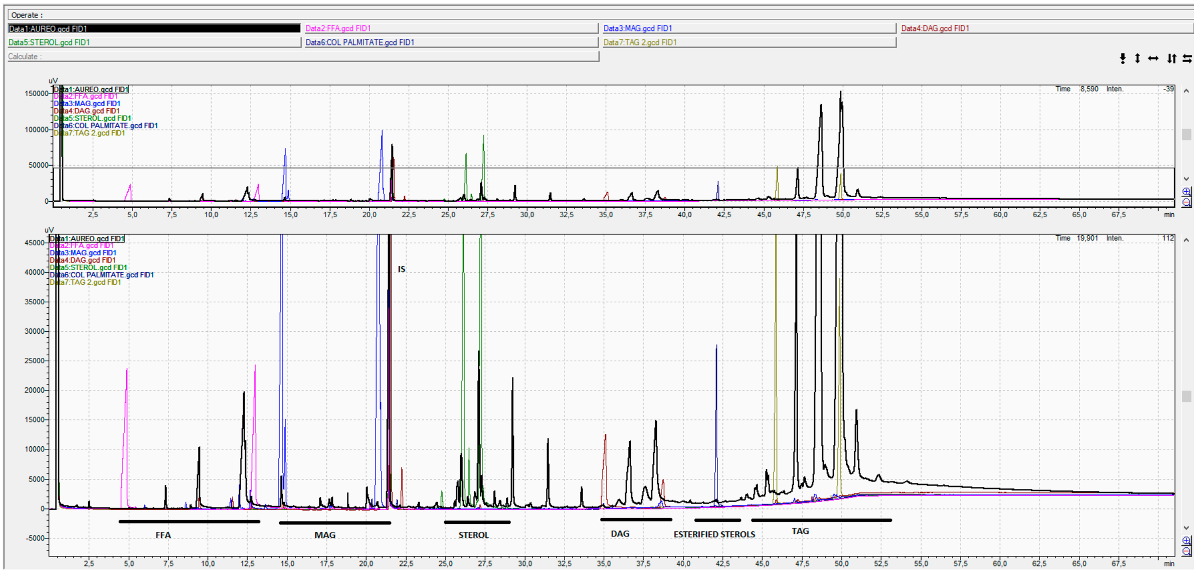

Single chromatograms of matrix

SAMPLE AUREO

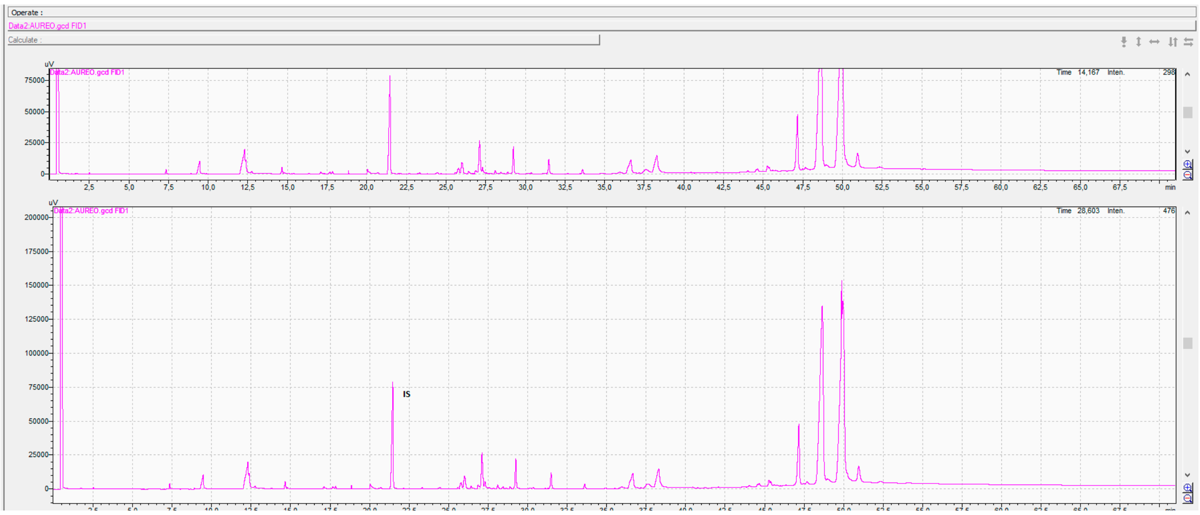

SAMPLE KRONOS

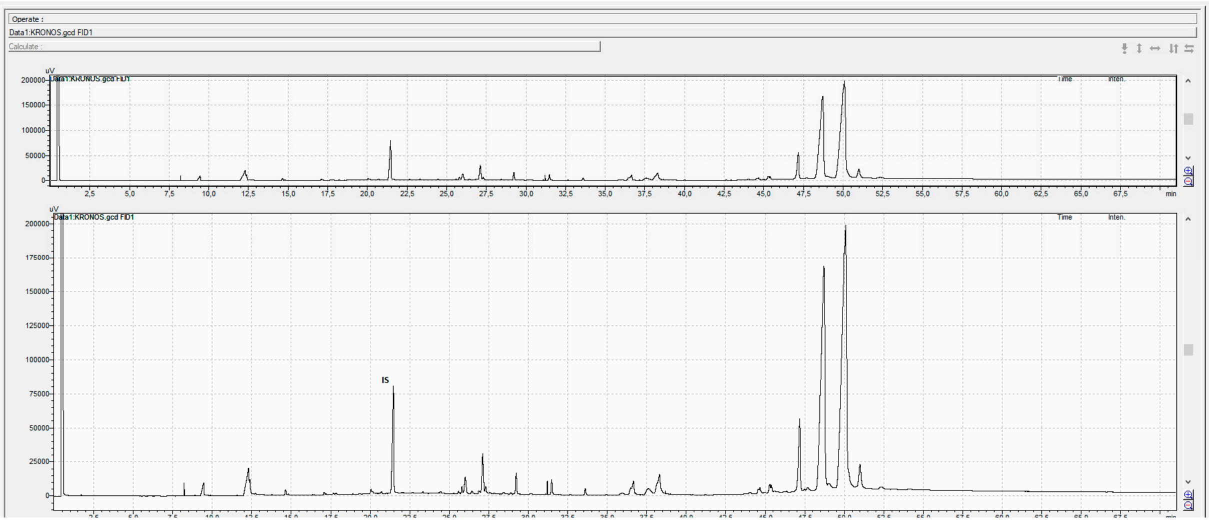

Single chromatograms standards

FFA

Tridecanoic acid; Stearic acid;

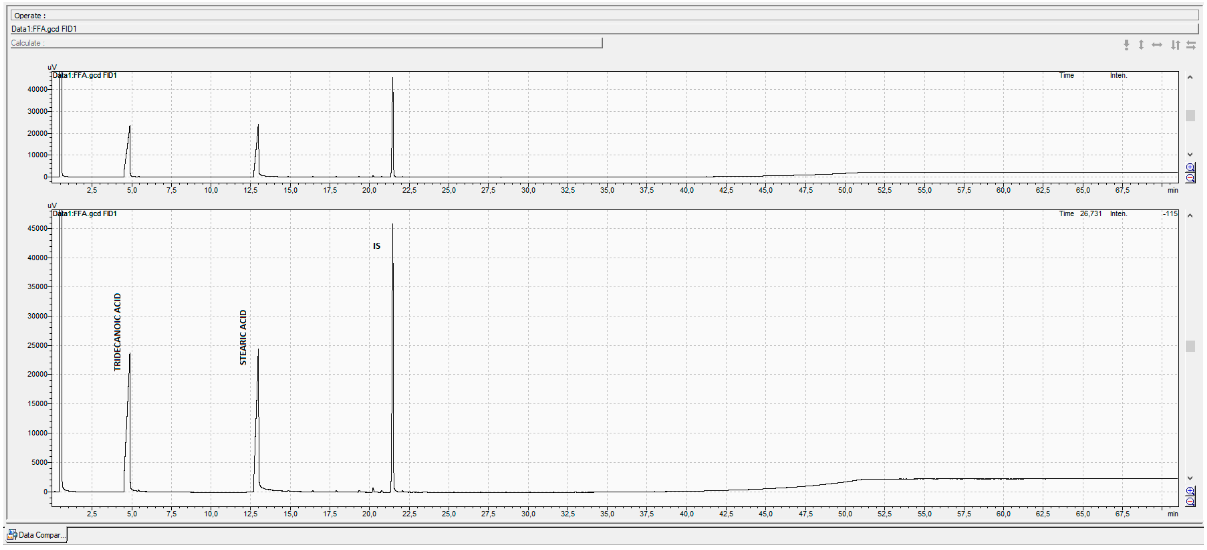

MAG

Monomiristin; Monostearin;

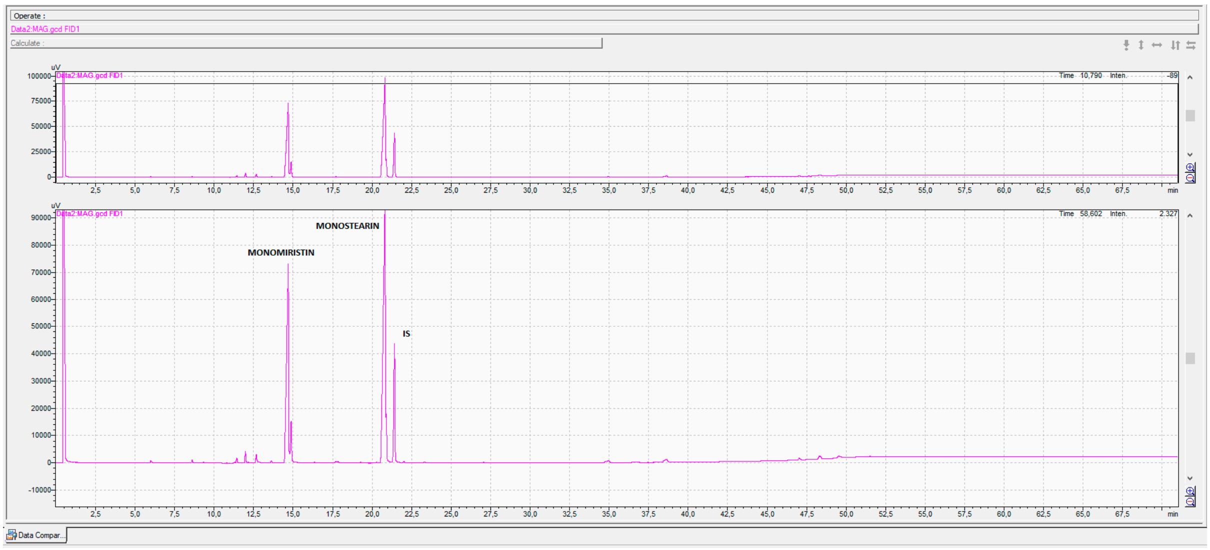

DAG
Dipalmitin; Distearin;

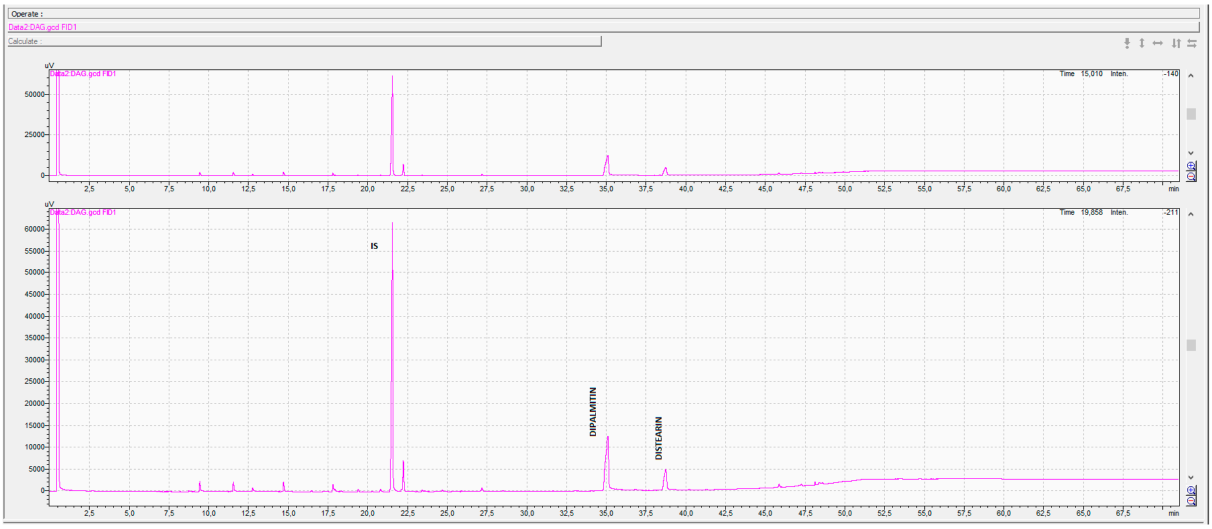

STEROL

B-Sitosterol 60%, also containing other sterols, primarily campesterol, was specially chosen.

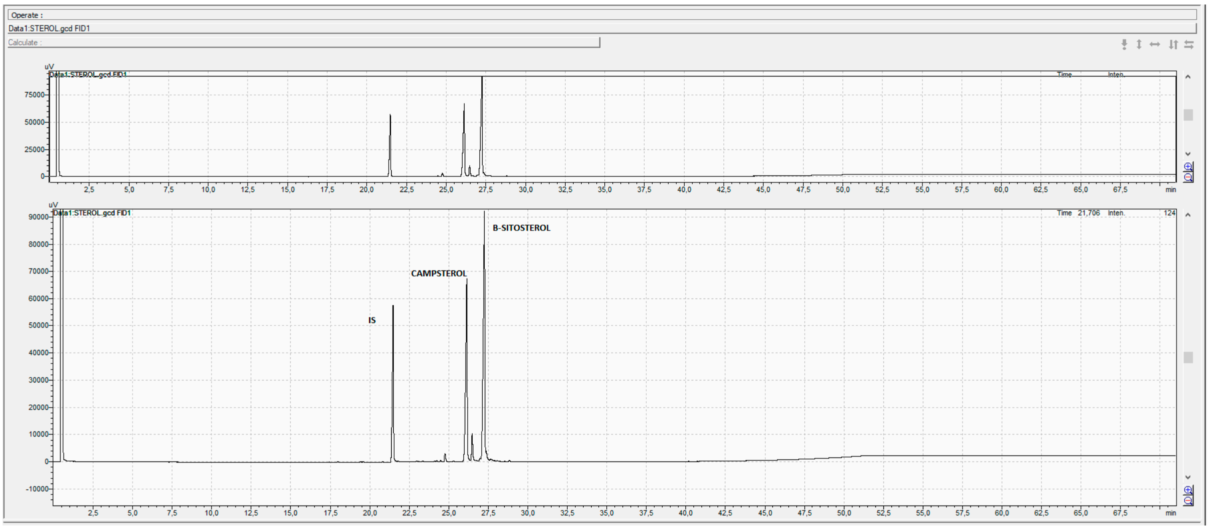

Cholesteryl palmitate;

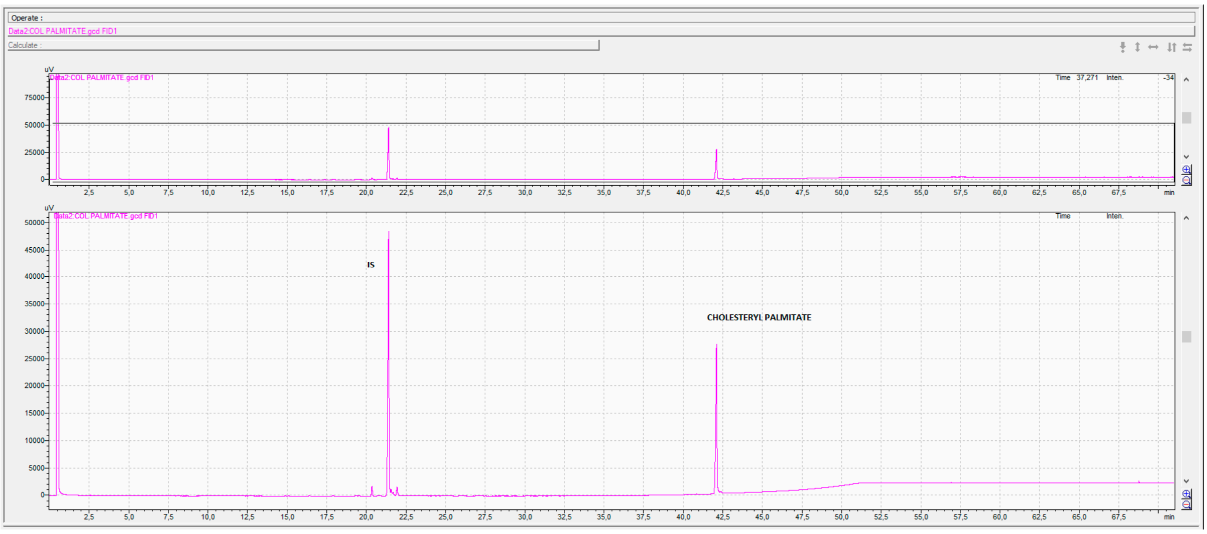

TAG

Tripalmitin; Tristearin;

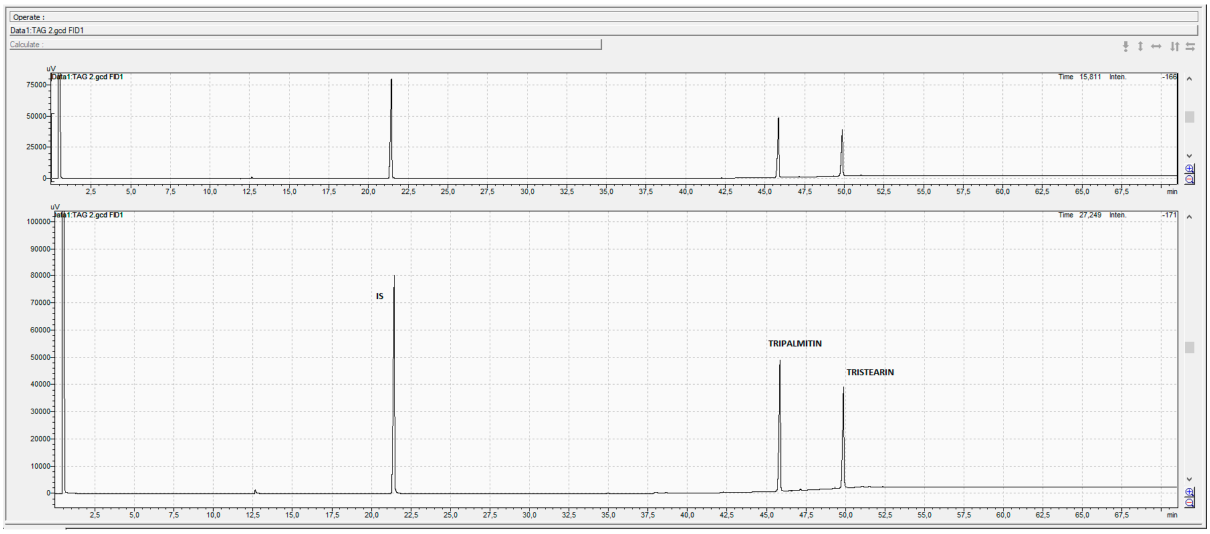

Supplement: Supplementary file 1 [file plants-13-01817-s001.zip › Supplementary Materials.pdf]
