# Supplementary material for: Lipids and Fatty Acid Composition Reveal Differences between Durum Wheat Landraces and Modern Cultivars
Source: Plants (Basel). 2024 Jul 1;13(13):1817. doi: 10.3390/plants13131817 (PMC11244281; doi:10.3390/plants13131817)
Supplement: Supplementary file 1 [file plants-13-01817-s001.zip › Supplementary Tables.pdf]

Supplementary Table S1: Heat map of Table 3. Main fatty acids composition and concentration (mg/100mg flour) in the kernel of the 22 wheat genotypes. Three technical replicate samples of each genotype were analysed. Colour ranking within columns.

	C12:0	C14:0	C15:0	C16:0	ΣC16:1t	C16:1	C17:0	C18:0	ΣC18:1	ΣC18:2t	C18:2	C20:0	ΣC20:1	C18:3	C20:2	C22:0	C22:1	C24:0	C24:1	C22:6
**Svevo**	0.02	0.09	0.10	13.56	0.10	0.16	0.07	1.47	14.98	0.13	44.93	0.15	0.66	4.08	0.08	0.13	0.08	0.16	0.06	0.04
**Iride**	0.01	0.06	0.10	12.53	0.08	0.17	0.06	0.89	15.38	0.10	45.58	0.12	0.69	4.17	0.09	0.14	0.08	0.17	0.07	0.04
**Odisseo**	0.01	0.06	0.09	12.44	0.08	0.14	0.06	1.00	13.29	0.12	43.28	0.12	0.59	4.67	0.09	0.14	0.07	0.17	0.06	0.04
**Monastir**	0.01	0.07	0.09	14.41	0.07	0.14	0.06	1.03	13.75	0.11	42.28	0.11	0.67	3.81	0.09	0.12	0.08	0.16	0.07	0.04
**Marco Aurelio**	0.01	0.06	0.09	13.14	0.07	0.16	0.06	0.82	14.16	0.11	43.86	0.12	0.71	3.28	0.10	0.12	0.09	0.14	0.07	0.05
**Aureo**	0.01	0.06	0.09	13.02	0.07	0.16	0.06	1.02	14.22	0.11	42.77	0.14	0.73	4.00	0.09	0.15	0.10	0.17	0.07	0.04
**Saragolla**	0.01	0.07	0.10	14.03	0.07	0.16	0.07	1.08	13.96	0.13	43.75	0.13	0.62	3.98	0.10	0.14	0.08	0.20	0.07	0.06
**Daurur**	0.01	0.06	0.08	12.54	0.07	0.11	0.06	1.01	14.35	0.12	43.11	0.13	0.65	2.98	0.09	0.13	0.08	0.15	0.06	0.05
**Strongfield**	0.01	0.07	0.09	14.16	0.08	0.15	0.07	1.13	13.05	0.12	45.52	0.15	0.69	3.59	0.10	0.15	0.08	0.18	0.06	0.05
**Simeto**	0.01	0.07	0.11	12.35	0.08	0.17	0.07	0.95	13.00	0.13	45.24	0.14	0.68	4.44	0.12	0.15	0.09	0.19	0.08	0.04
**Neodur**	0.01	0.07	0.08	14.26	0.06	0.15	0.06	0.99	16.07	0.11	45.15	0.13	0.70	2.96	0.09	0.13	0.08	0.16	0.07	0.03
**Creso**	0.01	0.07	0.10	13.08	0.07	0.16	0.07	1.13	14.80	0.12	44.40	0.14	0.58	3.80	0.08	0.14	0.06	0.18	0.06	0.05
**Kronos**	0.01	0.05	0.08	12.34	0.07	0.13	0.06	1.08	15.46	0.11	46.60	0.14	0.78	3.53	0.09	0.13	0.09	0.17	0.08	0.04
**Cappelli**	0.03	0.09	0.11	13.86	0.08	0.18	0.07	1.12	13.91	0.14	43.51	0.13	0.65	3.83	0.08	0.14	0.08	0.19	0.07	0.07
**Trinakria**	0.01	0.07	0.13	14.33	0.08	0.16	0.07	1.22	14.34	0.13	42.09	0.14	0.60	3.92	0.08	0.15	0.07	0.21	0.07	0.06
**Russello**	0.01	0.07	0.07	13.47	0.06	0.18	0.06	0.82	15.81	0.12	46.63	0.12	0.73	3.69	0.09	0.12	0.09	0.14	0.07	0.03
**Haurani**	0.01	0.07	0.10	13.06	0.07	0.19	0.06	0.91	15.99	0.12	46.55	0.13	0.67	3.45	0.11	0.13	0.09	0.15	0.06	0.03
**Kyperunda**	0.01	0.07	0.08	12.51	0.07	0.11	0.07	0.92	14.57	0.13	46.92	0.13	0.68	2.86	0.09	0.13	0.09	0.15	0.08	0.04
**Kubanka**	0.01	0.05	0.08	13.92	0.06	0.18	0.07	1.04	17.29	0.12	44.64	0.13	0.65	3.38	0.07	0.11	0.08	0.15	0.08	0.04
**Tetra-ipk_814**	0.02	0.10	0.12	14.64	0.09	0.18	0.09	1.06	11.83	0.16	43.56	0.16	0.73	4.28	0.11	0.26	0.09	0.29	0.08	0.05
**Tetra-ipk_815**	0.02	0.10	0.12	14.50	0.08	0.17	0.09	1.03	11.87	0.17	43.35	0.17	0.71	4.03	0.11	0.17	0.10	0.24	0.09	0.09
**AG189**	0.01	0.08	0.09	13.30	0.09	0.14	0.07	0.75	11.53	0.14	44.84	0.15	1.08	4.74	0.13	0.18	0.26	0.19	0.14	0.04

Supplementary Table S2: Heat map of Table 4. **Nutritional characteristics of the 22 wheat genotypes. Unsaturated (SFA). monounsaturated (MUFA) and polyunsaturated (PUFA) fatty acids are reported (mg/100 mg of fat). The ω6/ω3. UFA/SFA and PUFA/SFA ratios and the atherogenic (AI) and thrombogenic (TI) indices are reported. Colour ranking within columns.**

	SFA (mg/100 mg)	MUFA (mg/100 mg)	PUFA (mg/100 mg)	ω6/ω3	PUFA/SFA	UFA/SFA	AI	TI
**Svevo**	15.75	16.03	49.27	10.9	3.13	4.17	0.21	0.35
**Iride**	14.09	16.47	49.97	10.85	3.55	4.74	0.19	0.31
**Odisseo**	14.09	14.24	48.19	9.21	3.42	4.45	0.2	0.31
**Monastir**	16.07	14.78	46.32	11.02	2.88	3.82	0.24	0.39
**Marco Aurelio**	14.57	15.27	47.39	13.23	3.25	4.32	0.21	0.35
**Aureo**	14.7	15.34	47.01	10.6	3.2	4.26	0.21	0.34
**Saragolla**	15.83	14.97	48.03	10.85	3.03	4	0.23	0.36
**Daurur**	14.16	15.31	46.34	14.25	3.27	4.37	0.21	0.35
**Strongfield**	16.01	14.11	49.38	12.54	3.08	3.98	0.23	0.38
**Simeto**	14.02	14.09	49.97	10.13	3.56	4.59	0.2	0.31
**Neodur**	15.89	17.13	48.33	15.13	3.04	4.14	0.22	0.38
**Creso**	14.91	15.72	48.44	11.57	3.25	4.32	0.21	0.34
**Kronos**	14.06	16.6	50.36	13.11	3.58	4.78	0.19	0.32
**Cappelli**	15.73	14.98	47.62	11.19	3.03	4	0.23	0.37
**Trinakria**	16.32	15.32	46.28	10.59	2.84	3.79	0.24	0.38
**Russello**	14.89	16.95	50.56	12.56	3.4	4.55	0.2	0.33
**Haurani**	14.61	17.07	50.26	13.4	3.44	4.63	0.2	0.33
**Kyperounda**	14.06	15.61	50.04	16.2	3.56	4.69	0.2	0.34
**Kubanka**	15.56	18.34	48.26	13.07	3.1	4.3	0.21	0.36
**Tetra-ipk 814**	16.72	13	48.15	10.09	2.88	3.68	0.25	0.38
**Tetra-ipk 815**	16.43	13.01	47.75	10.55	2.91	3.72	0.25	0.38
**AG 189**	14.82	13.23	49.9	9.39	3.37	4.28	0.22	0.32
