# Supplementary figures and images for: Lipids and Fatty Acid Composition Reveal Differences between Durum Wheat Landraces and Modern Cultivars
Source: Plants (Basel). 2024 Jul 1;13(13):1817. doi: 10.3390/plants13131817 (PMC11244281; doi:10.3390/plants13131817)

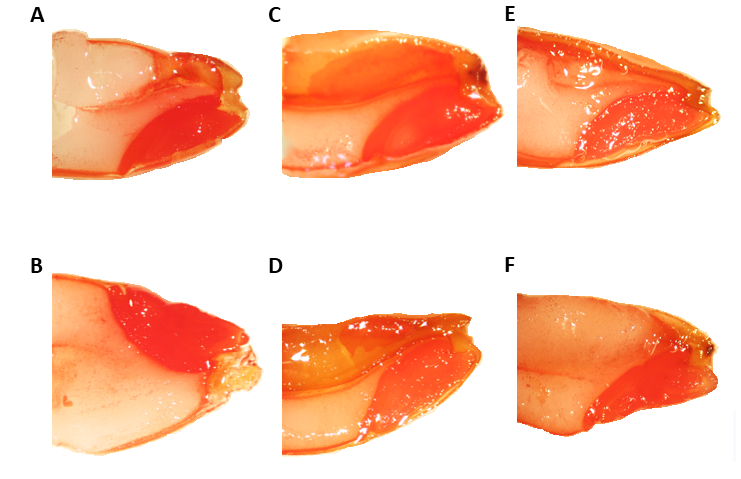

Supplement: Supplementary file 1 [file plants-13-01817-s001.zip › Supplementary Figure S1.tif]

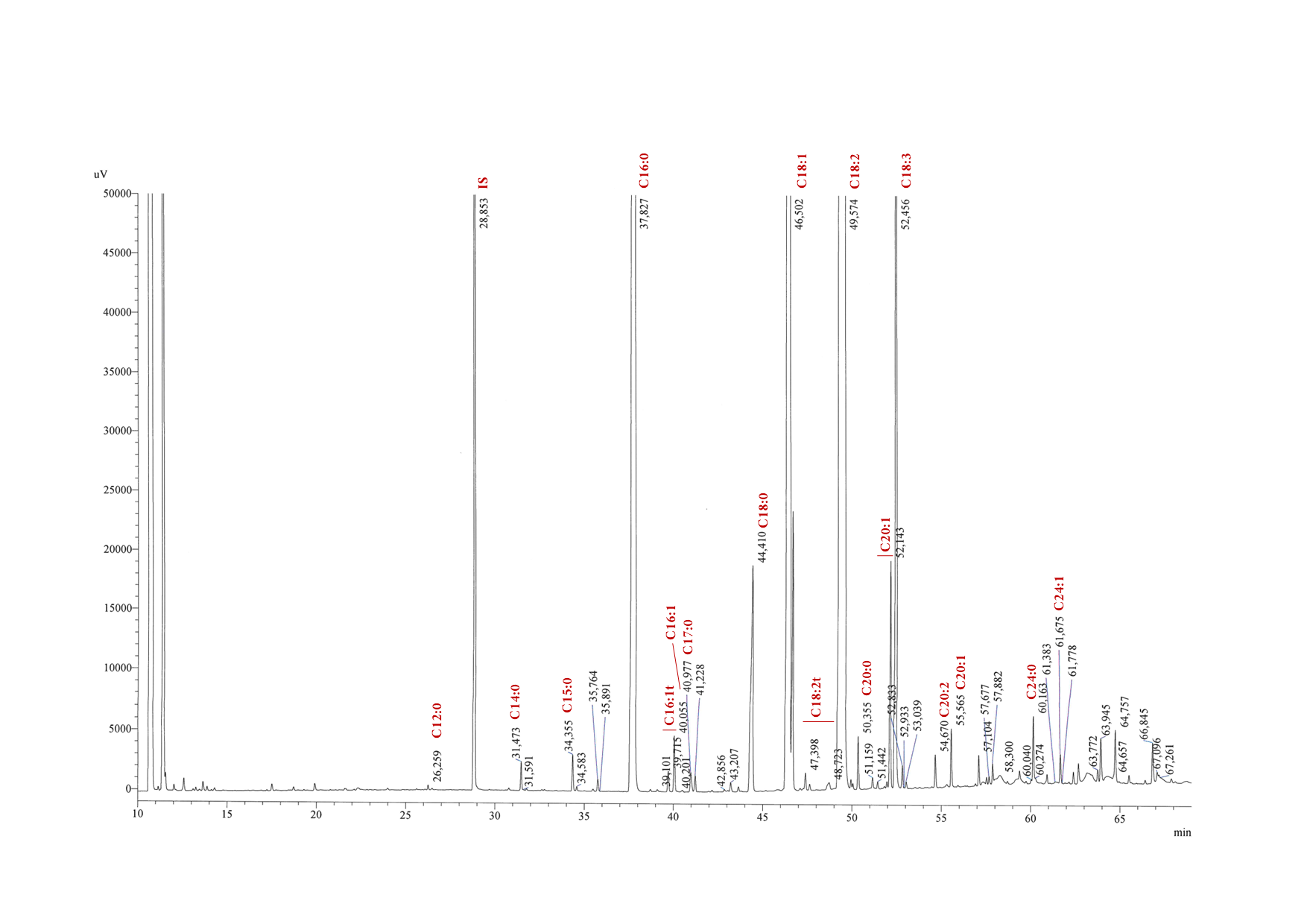

Supplement: Supplementary file 1 [file plants-13-01817-s001.zip › Supplementary Figure S2.tif]
